# Harnessing no-photon exciton generation chemistry to engineer semiconductor nanostructures

**DOI:** 10.1038/s41598-017-10751-x

**Published:** 2017-09-06

**Authors:** David Beke, Gyula Károlyházy, Zsolt Czigány, Gábor Bortel, Katalin Kamarás, Adam Gali

**Affiliations:** 1 Wigner Research Centre for Physics, Institute for Solid State Physics and Optics, Hungarian Academy of Sciences, P.O. Box 49, H-1525 Budapest, Hungary; 2 Faculty of Chemical Technology and Biotechnology, Budapest University of Technology and Economics, Műegyetem rkp. 7-9., H-1111 Budapest, Hungary; 3 Institute for Technical Physics and Materials Science, Centre for Energy Research, Hungarian Academy of Sciences, Konkoly-Thege M. út 29-33., H-1121 Budapest, Hungary; 4 Department of Atomic Physics, Budapest University of Technology and Economics, Budafoki út 8., H-1111 Budapest, Hungary

## Abstract

Production of semiconductor nanostructures with high yield and tight control of shape and size distribution is an immediate quest in diverse areas of science and technology. Electroless wet chemical etching or stain etching can produce semiconductor nanoparticles with high yield but is limited to a few materials because of the lack of understanding the physical-chemical processes behind. Here we report a no-photon exciton generation chemistry (NPEGEC) process, playing a key role in stain etching of semiconductors. We demonstrate NPEGEC on silicon carbide polymorphs as model materials. Specifically, size control of cubic silicon carbide nanoparticles of diameter below ten nanometers was achieved by engineering hexagonal inclusions in microcrystalline cubic silicon carbide. Our finding provides a recipe to engineer patterned semiconductor nanostructures for a broad class of materials.

## Introduction

There is urgent need for a simple and robust technology to fabricate molecular-sized semiconductor nanoparticles (NPs) and different types of nanostructures with large yield^1^. Electrochemical fabrication processes are technologically mature techniques^2^ to produce different types of nanostructures because of their simplicity and cost-competitiveness^3, 4^. Different variants of electrochemical fabrication processes might be categorized into active and passive methods. In active electrochemical methods either external bias or photo-excitation, or the combination of the two is applied to introduce charge carriers, electron – hole pairs or excitons that facilitate the chemical reactions leading to the dissolution of the material in the presence of etchants. In the passive electrochemical methods, either metal assisted or simple electroless wet chemical etching is applied. The latter is also called stain etching. Stain etching is the most advantageous technique as it can be employed to virtually any form of the material (powder, bulky or wafer), and no metal contacts, doping or illumination is required where the generality of the latter may be limited by the absorption cross section of the semiconductor and technological challenges caused by the harsh environment. However, the mechanism behind stain etching is largely unexplored that seriously limits its huge potential in fabrication of semiconductor nanostructures. For instance, wide band gap semiconductors, especially, silicon carbide^5^ and nitrides^6^ are exceedingly resistant against wet etchants.

**Figure 1:**
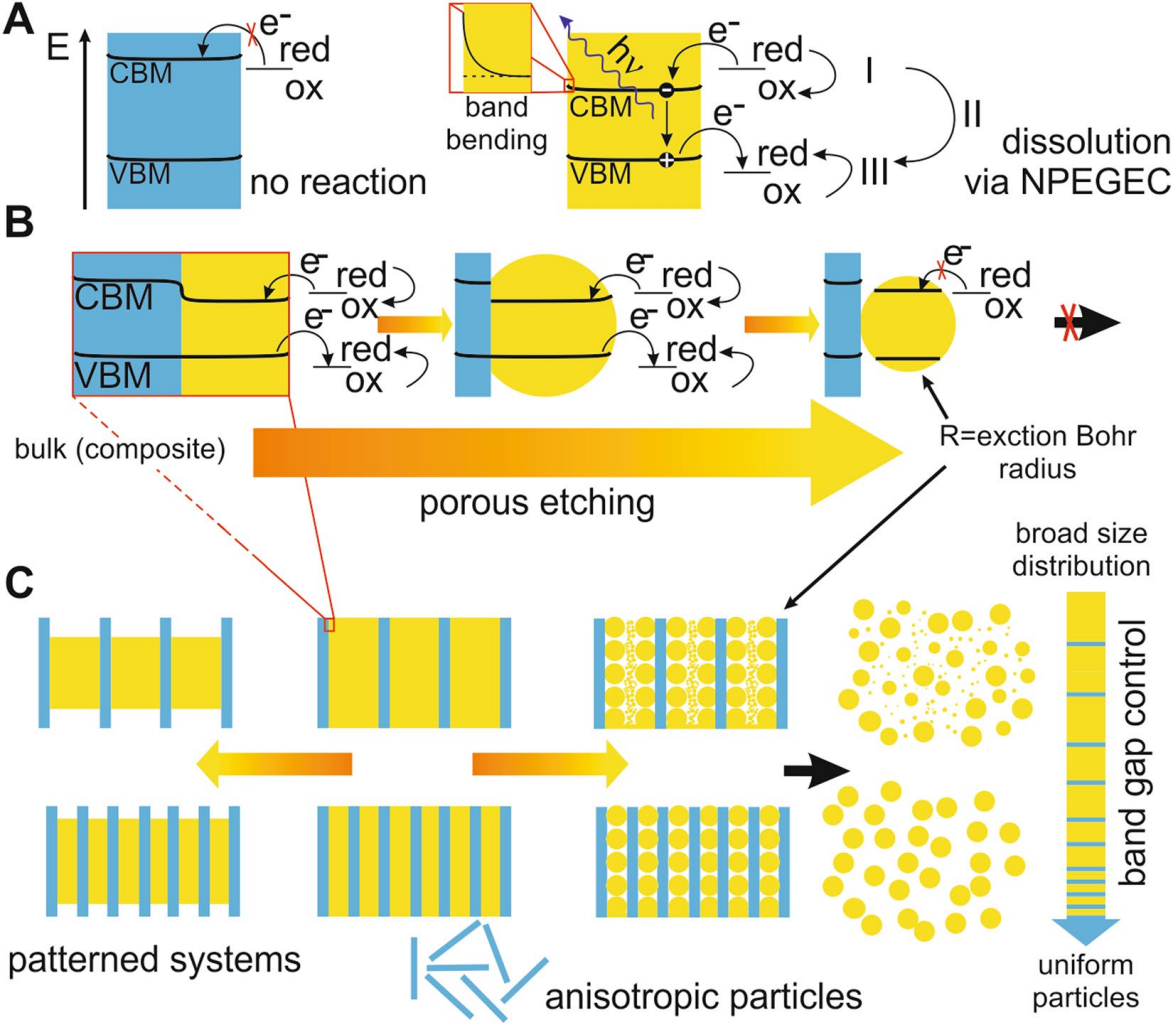
The mechanism “no-photon exciton generation chemistry” (NPEGEC) for stain etching of semiconductors. (**A**) The blue region depicts a semiconductor with a larger band gap that is resistive against etching while the yellow region represents a suitable material. A redox couple with redox potential higher (more negative) than the conduction band minimum (CBM) energy can inject electrons into the conduction band (I). The oxidized molecule itself, or the molecule formed after further transformation in the solution (II) can inject holes into the valence band (VB) with a maximum energy of VBM (III). The generated excitons can recombine with photon emission with energy *h*ν or can lead to material dissolution. **(B)** In a material with spatially varying band structure selective etching is possible. The exciton Bohr radius limits the radius (R) of the final nanoparticle. **(C)** Patterned band structure in a macroscopic material can serve as a template for various nanostructures including patterned nanowires, anisotropic or uniform particles.

Stain etching is usually described as an oxidation process. By forming a contact between the semiconductor surface and an electrolyte it is assumed^7^ that a hole is injected into the valence band (VB), close to the interface, by a strong oxidant in the electrolyte. The presence of a hole in the VB reduces the strength of bonds in its vicinity and makes the substrate atoms susceptible to attack by nucleophiles that should be present in the solution too, and then it starts dissolution and pore formation of the semiconductor. The initiation of the electrochemical process requires hole injection into VB in this model. Here we demonstrate that electron injection to the conduction band (CB), a reduction step^8^, is rather a predominant factor in the initiation of stain etching that facilitates chemical reactions in the solution that creates a strong oxidizing agent^9^ that finally leads to hole injection into VB (see Fig. 1A). As a consequence, the interaction of the semiconductor surface with the solution leads to exciton generation without illumination or external bias by this multistep electrochemical process. We call this multistep process no-photon exciton generation chemistry (NPEGEC). In NPEGEC, electron injection is the crucial step, therefore, the etching process only starts when the conduction band minimum (CBM) energy lies below the redox potential of a redox couple in the electrolyte. This finding can be used to seek suitable etchants for such semiconductors where their dissolution has not yet been achieved so far. By understanding the nature of stain etching, one can design experiments with engineered macroscopic materials to produce many different nanostructures like patterned wires, anisotropic or monodisperse nanoparticles (Fig. 1C). Monodisperse nanoparticles may be achieved by stain etching when the average distance between the electron blocking layers (blue region with enhanced CBM energy) is about the twice the exciton Bohr radius of the etched material (yellow region in Fig. 1B,C). This is caused by the size-dependent band bending effect in nanosized semiconductors^10, 11^ (see Supplemental Materials for details).

Silicon carbide (SiC) is an excellent model material to demonstrate NPEGEC and its application for nanoparticle engineering. SiC can crystallize in different polymorphs called polytypes with very different band gaps^12^ but the same Si-C binding energies (see Supplemental Materials). Therefore the same chemical reactions are expected to occur on all polytypes. However, the cubic type of the material can be attacked with hot HF:HNO_3_ solutions leading to thin pore formation on the surface^13, 14^ while other polytypes are resistive to such chemicals. The reason for this selectivity has not been understood. We discuss this phenomenon by NPEGEC in stain etching. The VBM of SiC polytypes resides at +1.5 V vs SHE (standard hydrogen electrode)^15^ which is considerably higher (more positive) than the redox potential of nitric acid +0.88 ± 0.05 V vs SHE for the possible redox couples^9, 16^ or of the well known active species, NO^+^. NO^+^ has a redox potential of +1.45 ± 0.05 V vs SHE^17^, thus the pore formation of cubic SiC polytype should not occur at all by considering only hole injection as a driving force behind the pore formation. However, NPEGEC explains these observations. We suggest the nitrosyl ion (NO^−^), generated in the solution^18, 19^ as one of the active agents in stain etching of cubic SiC. NO^−^ is a very reactive species and the redox potential of NO^−^/NO is around −0.9 V vs SHE^9, 20^ that is above the CBM energy of cubic SiC but below the CBM energy of hexagonal polytypes (see Supplemental Materials). Thus, NO^−^ is oxidized to neutral NO radical at the cubic SiC surface while it injects an electron into the CB. The resultant NO radicals can inject holes to the VB (the NO/N_2_O redox couple has a redox potential of +1.6 ± 0.05 V vs SHE^9^) that oxidizes cubic SiC (Fig. S3). Finally, the nucleophile, HF, is able to dissolve this material. In the most common hexagonal polytypes, 6H and 4H (see Supplementary Materials), the CBM energies lie 0.4 eV–1.5 eV above the redox potential of NO^−^/NO. As a consequence, the formation of NO radicals is hindered so the oxidation process does not occur. We note that the lifetime of the NO radical is very short at elevated temperatures^21^, and it reacts very efficiently with many molecules in the solution^22^. As a consequence, the vast majority of NO radicals can be only found at the surface of 3C-SiC in the etching process, thus only the 3C-SiC part of a mixed cubic/hexagonal SiC can be etched efficiently.

**Figure 2:**
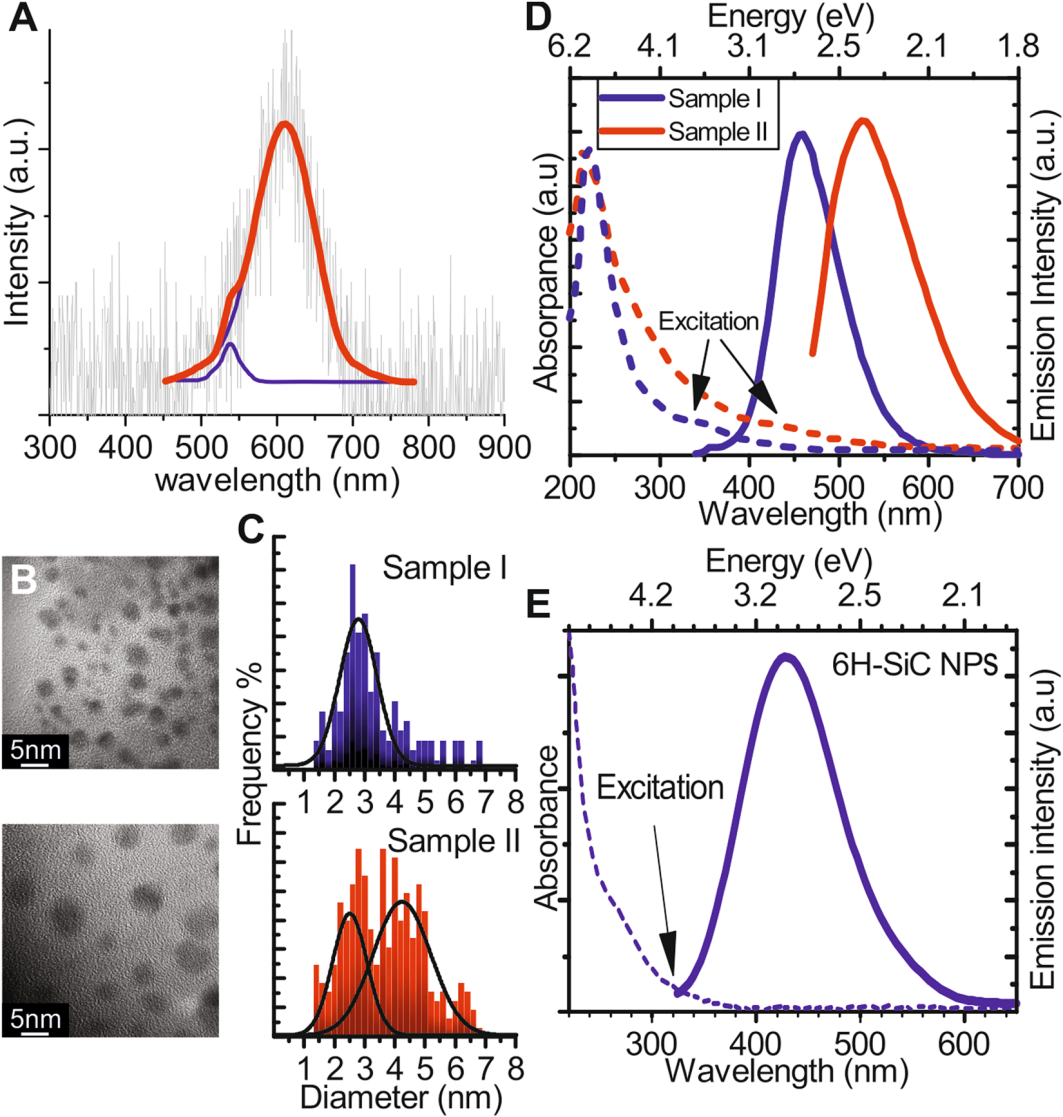
Characterization of SiC particles prepared from different SiC sources. (**A**) The measured chemiluminescence during stain etching of SiC. (**B**) TEM images of nanoparticles prepared from pure 3C-SiC (sample I), 3C-SiC with 15% fraction of hexagonal inclusions (sample II). (**C**) Size distribution of samples I and II ﻿﻿﻿which was taken from more than 300 NPs that were observed in several different ﻿TEM images. (**D**) UV-VIS absorption and emission of samples I and II. Sample II contains larger particles, therefore, the luminescence is red-shifted^31^. (**E**) UV-VIS absorption and emission spectra of 6H-SiC nanostructures.

We verify the CBM mediated mechanism of stain etching on SiC polytypes. First, we study the presence of NO^−^ in the hot HF:HNO_3_ solution. NO^−^ is known to rapidly reduce Cu^(II)^ ions^23^, therefore, the presence of Cu^(II)^ ions in the solution could introduce competitive reactions with SiC^24^. This should lead to substantial reduction of the etching process. Indeed, by adding CuF_2_ to the HF:HNO_3_ etchant, the resultant Cu^(II)^ stopped the nanoparticle formation (see Supplemental Materials). Second, the presence of excitons in the etching process is demonstrated. If electrons and holes are simultaneously injected by chemical processes then these generate excitons that may recombine either non-radiatively by phonons or Auger-processes, or radiatively by emitting photons, where the latter can be detected by photodetectors. Indeed, we observe luminescence during stain etching in dark without applying any external bias (see Fig. 2A). The emission exhibits a maximum at around 610 nm with a shoulder at 535 nm. The emission with a maximum at around 610 nm was already reported in previous studies on porous 3C-SiC using different excitation methods associated with the surface defects of SiC created by etching^25–28^. As our etching continuously creates porous SiC, a similar spectrum may be expected when the band-edge-to-defect-level optical transition is associated with this luminescence spectrum. The wavelength of the other emission center coincides with the indirect band gap of 3C-SiC (see Supplemental Materials), that we associate with the phonon mediated emission between the band edges of 3C-SiC. This experimental fact implies that free electrons and holes are generated during the etching process. We note that the etchants themselves do not produce such a chemilumenscence spectrum^29^. We further note that the chemiluminescence spectrum of surface related defects implies an occupied or empty defect level in the fundamental band gap of 3C-SiC that resides around 0.3 eV with respect to either the VBM or the CBM, respectively. We did not find any evidence about the role of surface defect states in the etching process. Nevertheless, if they play an active role then they would not change the NPEGEC model but slightly alter the effective value of the “VBM” or the “CBM” of 3C-SiC.

The selectivity of NPEGEC can be used to fabricate different types of nanostructures by engineering 3C/6H-SiC heterostructures. The polytype selectivity was already shown on patterned SiC nanowires^13^ where the selectivity of such etchant was used to dissolve 3C-SiC from 3C-SiC/6H-SiC matrix (see Figs 1C and S3). While patterning in a selective etching process is evident, we propose here, that the alternation of the CBM energy in a macroscopic system can be also used to tune the size distribution of nanoparticles prepared by stain etching (Fig. 1B). Hexagonal inclusions in cubic SiC act as blocking layers for CB electrons that can quasi homogeneously appear in the bulk cubic matrix. These hexagonal inclusions are stacking faults (SF) in 3C-SiC that may be described as small hexagonal polytype inclusions (see Fig. S1). As the SF concentration increases, the distance between two SFs decreases. If the distance between SFs approaches twice the exciton Bohr radius then the etching will stop due to the size-dependent band bending of the semiconductor particles (see Fig. S3 and the text in the Supplementary Materials). As a consequence, the population of SiC NPs with size close to twice the exciton Bohr radius will dominate with forming a quasi monodisperse distribution (see Fig. 1B,C). Particularly, by assuming that SFs are uniformly distributed and ordered to form small 4H-SiC inclusions consisting of one cubic bilayer sandwiched by two hexagonal bilayers (see Fig. S1) then 15% SF concentration shall lead to an average distance of 5 nm between two SFs that is twice the exciton Bohr radius in 3C-SiC. At this condition, the diameter of the etched SiC NPs should be around 5 nm.

To demonstrate such phenomenon, we synthesized 3C-SiC powder with about 15% concentration of hexagonal inclusions by a robust and high output method (see Fig. S2 and the text in the Supplementary Materials) and we prepared nanoparticles from them by etching. The differences in size distribution of NPs produced from different SiC sources can be readily observed on the transmission electron microscopy (TEM) images (Fig. 2B). Figure 2 also shows the size distribution (Fig. 2C), and optical properties of SiC NPs (Fig. 2D) that were prepared from SiC powder of 0% (sample I) and 15% (sample II) SF fractions, respectively. Sample I contains 1–4 nm particles with emission at around 450 nm^30^. Sample II contains a significant amount of 4–6 nm particles (Fig. 2B,C) and shows a redshift in the luminescence (Fig. 2D)^30^. We note that we define SF fraction as diffracted intensity fraction attributed to inhomogeneities in the crystal which correlate with the SF concentration (see Fig. S2A). However, SFs in our 3C-SiC microcrystal are not evenly distributed and do not always form 4H-SiC inclusions that leads to variation of distances between SFs. As a result Sample II still contains a significant number of particles with diameter less than twice the exciton Bohr radius. Nevertheless, this finding clearly demonstrates (see also Fig. S4) that the size of the fabricated SiC NPs can be controlled by varying SF concentration, i.e., polytype inclusions in the SiC microcrystal.

Finally, we used the mechanism of NPEGEC to find suitable etchant for 6H-SiC, too. The dithionate (S_2_O_6_^2−^) ion decomposes above 70 °C^32^ and various ions and radicals can form^33, 34^ including SO_3_^2−^ with a reduction potential of −1.36 ± 0.24 V at the SO_3_^2−^/S_2_O_4_^2−^ redox couple^35^ that can inject electrons into the CB of 6H-SiC. Figure 2E shows the absorption and emission spectra of 6H-SiC NPs made by NPEGEC etching of 6H-SiC in HF:K_2_S_2_O_6_ electrolyte. The emission is similar to other polytype NPs^36, 37^ because of the surface state related emission^38^, however, the absorption edge is blue shifted from 450 nm of 3C-SiC to 400 nm of 6H-SiC because of the enlarged band gap of the 6H polytype.

**Figure 3:**
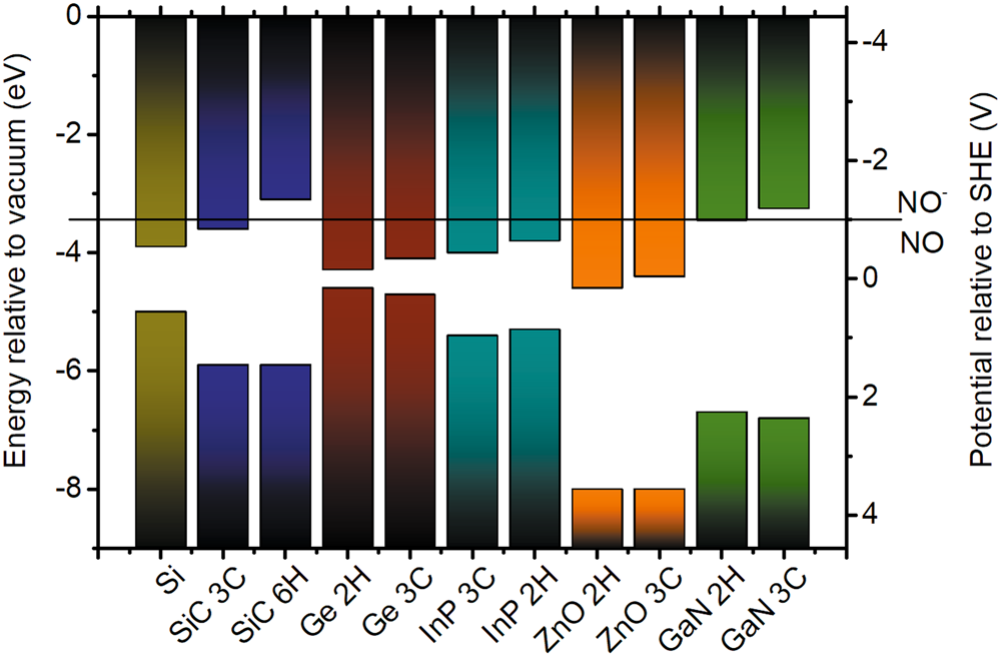
Alignment of band edge positions for semiconductors with different polytypes is depicted where the horizontal line represents the level vs. standard hydrogen electrode (SHE) of the redox potential of NO/NO^−^ which is a strong reducing agent in HNO_3_ etchant^43^. References about the data of band edges can be found in the Supplementary Materials.

Polytypism is known for many other compound semiconductors. Particularly, during the growth of semiconductor nanowires, polytype inclusions were identified^39, 40^. These polytype inclusions alter the band edge energies similarly to the polytypes of SiC. Our findings on stain etching provide a method to find suitable etchants that have redox potentials between the CB energies of the corresponding polytypes, in order to realize polytype selective etching. This effect leads to the formation of patterned nanostructures or NPs. In Fig. 3 we plotted the VBM and CBM energies of technologically important semiconductors at pH 0 that exist in different polytypes (at least, in the form of nanowires) and suitable for selective etching. HNO_3_ etchant selectively etches polymorphs of bio- and hemocompatible SiC^41, 42^ but other etchants may be chosen for other materials where the preselection should be based on the corresponding redox potentials (see the database in ref. 16). Our finding paves the way toward the design of etching strategies for efficient production of semiconductor nanoparticles that are applied in diverse areas such as chemical and electrical sensors, photovoltaics, or quantum electronic devices that may accelerate the scientific research and technological advance in a wide range of fields.

## Electronic supplementary material

Supplementary Materials

## References

[1] Sequeira CAC, Santos DMF (2009). Electrochemical routes for industrial synthesis. J. Braz. Chem. Soc..

[2] Chang W (2004). Micromachining of p-type 6H-SiC by electrochemical etching. Sensors Actuators A Phys..

[3] Santinacci L, Djenizian T (2008). Electrochemical pore formation onto semiconductor surfaces. Comptes Rendus Chim..

[4] Urmann, K., Tenenbaum, E., Walter, J. G. & Segal, E. *Electrochemically Engineered Nanoporous Materials*. *Springer Series in Materials Science* **220**, (2015).

[5] Zhuang D, Edgar JH (2005). Wet etching of GaN, AlN, and SiC: a review. Mater. Sci. Eng. R Reports.

[6] Kallesøe C (2008). Selective etching of III–V nanowires for molecular junctions. Microelectron. Eng..

[7] Ayat M (2014). Formation of nanostructured silicon surfaces by stain etching. Nanoscale Res. Lett..

[8] Yong X, Schoonen MAA (2000). The absolute energy positions of conduction and valence bands of selected semiconducting minerals. Am. Mineral..

[9] Dutton AS, Fukuto JM, Houk KN (2005). Theoretical Reduction Potentials for Nitrogen Oxides from CBS-QB3 Energetics and (C)PCM Solvation Calculations. Inorg. Chem..

[10] Hagfeldt A, Graetzel M (1995). Light-Induced Redox Reactions in Nanocrystalline Systems. Chem. Rev..

[11] Wang F, Buhro WE (2007). Determination of the rod-wire transition length in colloidal indium phosphide quantum rods. J. Am. Chem. Soc..

[12] van de Lagemaat J, Vanmaekelbergh D, Kelly JJ (1998). Photoelectrochemical characterization of 6H–SiC. J. Appl. Phys..

[13] Cambaz GZ, Yushin GN, Gogotsi Y, Lutsenko VG (2006). Anisotropic etching of SiC whiskers. Nano Lett..

[14] Beke D (2013). Preparation of small silicon carbide quantum dots by wet chemical etching. J. Mater. Res..

[15] Memming RR (1994). Photoinduced charge transfer processes at semiconductor electrodes and particles. Electron Transf. I.

[16] Allen, J. B., Parsons, R. & Joseph, J. *Standard Potentials in Aqueous Solution*. (CRC press, 1985).

[17] Kelly MT, Chun JKM, Bocarsly AB (1994). High efficiency chemical etchant for the formation of luminescent porous silicon. Appl. Phys. Lett..

[18] Steinert M, Acker J, Krause M, Oswald S, Wetzig K (2006). Reactive species generated during wet chemical etching of silicon in HF/HNO3 mixtures. J. Phys. Chem. B.

[19] Janaway GA, Zhong M, Gatev GG, Chabinyc ML, Brauman JI (1997). [FHNO] -: An Intermediate in a Spin-Forbidden Proton Transfer Reaction. J. Am. Chem. Soc..

[20] Miranda KM (2005). The chemistry of nitroxyl (HNO) and implications in biology. Coord. Chem. Rev..

[21] Matsumoto K (2011). Temperature-dependent free radical reaction in water. J. Clin. Biochem. Nutr..

[22] Lide, D. D. Electrochemical Series. *CRC Handb. Chem. Physics, 87th Ed*. 1–10, doi:10.1136/oem.53.7.504 (2005).

[23] Nelli S, Hillen M, Buyukafsar K, Martin W (2000). Oxidation of nitroxyl anion to nitric oxide by copper ions. Br. J. Pharmacol..

[24] Spoto G, Bordiga S, Scarano D, Zecchina A (1992). Well defined CuI(NO), CuI(NO)2 and CuII(NO)X (X = O− and/or NO2−) complexes in CuI-ZSMS prepared by interaction of H-ZSM5 with gaseous CuCl. Catal. Letters.

[25] Monguchi T (1998). Structural and Optical Characterization of Porous 3C-SiC. J. Electrochem. Soc..

[26] Zhi-ming C, Jian-ping M, Jian-nong W (1999). Photoluminescence from Porous-Like SiC and Its Light-Induced Enhancement Photoluminescence. Chinese Phys. Lett..

[27] Konstantinov AO, Henry A, Harris CI, Janzén E (1995). Photoluminescence studies of porous silicon carbide. Appl. Phys. Lett..

[28] Liu L, Yiu YM, Sham TK, Zhang L, Zhang Y (2010). Electronic Structures and Optical Properties of 6H- and 3C-SiC Microstructures and Nanostructures from X-ray Absorption Fine Structures, X-ray Excited Optical Luminescence, and Theoretical Studies. J. Phys. Chem. C.

[29] Sehgal C, Sutherland RG, Verrall RE (1980). Sonoluminescence of nitric oxide- and nitrogen dioxide-saturated water as a probe of acoustic cavitation. J. Phys. Chem..

[30] Beke D, Szekrényes Z, Czigány Z, Kamarás K, Gali Á (2015). Dominant luminescence is not due to quantum confinement in molecular-sized silicon carbide nanocrystals. Nanoscale.

[31] Dohnalová K (2013). Surface brightens up Si quantum dots: direct bandgap-like size-tunable emission. Light Sci. Appl..

[32] Lente G, Fábián I (2004). Effect of dissolved oxygen on the oxidation of dithionate ion. Extremely unusual kinetic traces. Inorg. Chem..

[33] Liang C, Su HW (2009). Identification of sulfate and hydroxyl radicals in thermally activated persulfate. Ind. Eng. Chem. Res..

[34] Hemmingsen T (1992). The electrochemical reaction of sulphur-oxygen compounds-part I. A review of literature on the electrochemical properties of sulphur/sulphur-oxygen compounds. Electrochim. Acta.

[35] Dutta SK, Ferraudi G (2001). Mechanism of Redox Reactions between SO 3 •- Radicals and Transition-Metal Macrocyclic Complexes: Oxidative Addition to the Ligand and Outer-Sphere Electron Transfer. J. Phys. Chem. A.

[36] Botsoa J (2009). Luminescence mechanisms in 6H-SiC nanocrystals. Phys. Rev. B.

[37] Fan J, Li H, Wang J, Xiao M (2012). Fabrication and photoluminescence of SiC quantum dots stemming from 3C, 6H, and 4H polytypes of bulk SiC. Appl. Phys. Lett..

[38] Beke D (2016). Identification of Luminescence Centers in Molecular-Sized Silicon Carbide Nanocrystals. J. Phys. Chem. C.

[39] Caroff P (2009). Controlled polytypic and twin-plane superlattices in III-V nanowires. Nat. Nanotechnol..

[40] Glas F, Harmand JC, Patriarche G (2007). Why does wurtzite form in nanowires of III-V zinc blende semiconductors?. Phys. Rev. Lett..

[41] Schettini, N., Jaroszeski, M. J., West, L. & Saddow, S. E. In *Silicon Carbide Biotechnology* 153–208, doi:10.1016/B978-0-12-385906-8.00005-2 (Elsevier, 2012).

[42] Beke D (2013). Silicon carbide quantum dots for bioimaging. J. Mater. Res..

[43] Hammerl, A. & Klapötke, T. M. In *Encyclopedia of Inorganic and Bioinorganic Chemistry* 1–9 doi:10.1002/9781119951438.eibc0147 (John Wiley & Sons, Ltd, 2011).

