# Supplementary material for: Harnessing no-photon exciton generation chemistry to engineer semiconductor nanostructures
Source: Sci Rep. 2017 Sep 6;7:10599. doi: 10.1038/s41598-017-10751-x (PMC5587652; doi:10.1038/s41598-017-10751-x)
Supplement: Supplementary file 1 — Supplementary Materials [file 41598_2017_10751_MOESM1_ESM.pdf]

Supplementary Materials for

Harnessing no-photon exciton generation chemistry to engineer semiconductor nanostructures

Authors: David Beke[1*], Gyula Károlyházy[1,2], Zsolt Czigány[3], Gábor Bortel[1], Katalin Kamarás[1], Adam Gali[1,4*]

Materials and Methods:

Polytypes and stacking faults in SiC Similar to diamond and silicon, silicon carbide (SiC) is a sp^3 hybridized covalent system with tetrahedral bonds. SiC may form the well-known zinc blende structure with a face centered cubic (fcc) lattice. Along the (111) lattice direction Si–C bilayers can be found where the stacking sequences follow an fcc type close-packed structure (see Figure S1), and the periodicity can be described by three cubic (*k*) bilayers along that direction. Thus, cubic (C) SiC is called 3C-SiC in the context. However, it is known that it is possible to form a hexagonal close-packed (hcp) structure from the tetrahedrons that leads to hexagonal (*h*) stacking sequence. The crystal structure formed this way is the wurtzite lattice which is a hexagonal (H) lattice and two *h* bilayers provide the periodicity of that lattice, called 2H briefly. In SiC, different combination of stacking sequences of cubic and hexagonal bilayers can form different crystal structures, or polymorphs, that are common in the basal hexagonal plane. These special polymorphs are also called polytypes, and the crystal axis of the hexagonal polytype is conventionally called *c*-axis [1]. The most common polytypes are 4H and 6H with *hkhk* and $hk_1k_2hk_1k_2$ stacking sequences along the *c*-axis, respectively (see Fig. S1). The stacking of the bilayers may be also labeled as A, B and C, respectively. 3C-SiC can be described by the sequences of …ABC|ABC…, whereas 4H-SiC and 6H-SiC can be viewed as …ABCB|ABCB… and …ABCACB|ABCACB… sequences, respectively. The bandgaps of 3C, 6H and 4H

polytypes are 2.4, 3.0, and 3.3 eV, respectively [2]. This is a large variance despite the fact that the chemical Si-C bonds are the same in all of these polytypes. Regarding the variation of the bandgaps, the conduction band offset between the cubic and hexagonal polytypes is large while the valence band offset is minute[3]. Stacking faults in cubic SiC are hexagonal bilayers. As we illustrate in Figure S1 the hexagonal inclusion(s) in cubic SiC may be described as a small hexagonal polytype inclusion in cubic SiC that leads to the variation of the conduction band edge [4].

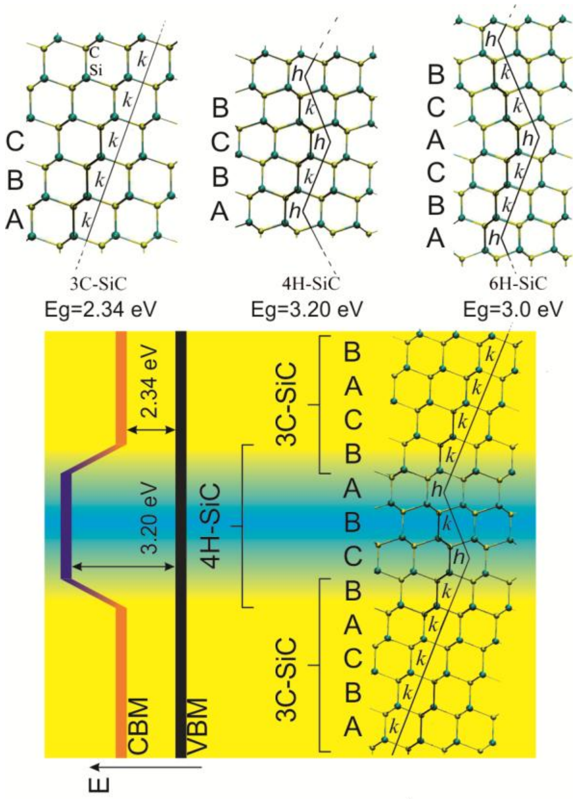

Fig. S1 Crystal structure and its nomenclature of the most common silicon carbide polytypes. The labels *k* and *h* depict the cubic and hexagonal stacking of Si-C bilayers along the (111) crystal direction of 3C-SiC or the (0001) direction in hexagonal SiC that is called *c*-axis. The stacking of bilayers can be also labeled by A, B and C, respectively. 4H-SiC inclusion is realized by inserting two hexagonal bilayers into the 3C-SiC crystal. The bandgap variation (*E*) of 4H-SiC inclusion embedded into 3C-SiC is depicted on the left where VBM and CBM labels the energy of the valence band maximum and conduction band minimum, respectively..

Synthesis of cubic SiC powder [5] Si (99%, 325 mesh, Sigma) and C [Norit A supra, surface area (BET), 1700 m^2/g) with 1:1 ratio and 3 wt% PTFE (Polytetrafluoroethylene) (1 µm particle size, Sigma) was mixed in a ball mill for 12 hours then placed into a graphite crucible. Samples were annealed up to about 1250 °C in argon atmosphere using an induction furnace. The heating procedure took 8 minutes then the furnace was switched off. The cooling process took about 20 minutes. The samples were then annealed at 750 °C in air for 10 hours to remove unreacted carbon, then $HF:HNO_3:H_2O$ 1:1:10 was used to remove unreacted Si and SiO_2.

We developed a technique to synthesize cubic SiC (3C-SiC) powder with varying hexagonal inclusions, then we applied stain etching on these SiC powders and studied the properties of the resultant SiC NPs. The hexagonal inclusions are stacking faults (SF) in cubic SiC that form hexagonal polymorphic or polytypic crystal inclusions. We emphasize that the hexagonal inclusions do not alter the inherent chemical bonding of the cubic morphology. These hexagonal polytypes have the VBM at the same energy within 0.05 eV but their CBM energy lies about 0.6-0.9 eV higher than that of the cubic SiC depending on the density of the hexagonal inclusions. We varied the amount of stacking fault (SF) in the cubic SiC host matrix with a variety of additives. Namely, boron, aluminum, nitrogen (as NH_4Cl), or PTFE was added to the Si:C:PTFE mixture. By increasing the additive concentration from 0.5 to 5 molar % of Al, N and B, or from 5 to 20 wt% of additional PTFE, the SF fraction was increased.

It should be noted that Al, B and N additives applied in the precursor are common acceptor and donor dopants of SiC. This might lead to varying Fermi level of SiC created in the presence of additives when these additives are incorporated into SiC in the heat combustion process. This possible scenario might also contribute to the measured size distribution of SiC NPs beside the detected SFs inside the cubic SiC. On the other hand, just by raising the PTFE concentration in

the precursor lead to an increased SF fraction with resulting in the same effect on the size distribution of SiC NPs, and PFTE does not contain any typical dopant of SiC. This rather implies that these other additives play the same role, i.e., increase of SF fraction in cubic SiC. Furthermore, we previously measured the additive concentration of Al by using atomic absorption spectroscopy and electron loss spectroscopy during TEM analysis. The Si/Al molar ratio for samples fabricated from precursor without Al is around 0.14 [6] and we did not measure any higher concentration of Al even when Al is added to the precursor. This data proves that no more Al is built in our nanoparticles than that prepared without Al in the precursor, and the predominant effect of Al in the precursor is the incorporation of hexagonal bilayers into cubic SiC.

Synthesis of 3C-SiC NPs 3C-SiC NPs were prepared from SiC powders via stain etching. Briefly[7], SiC was etched in HF:HNO_3 3:1 mixture at 150 °C using an acid digestion chamber (Berghoff GMBH) then the acid was removed and the powder was sonicated in water for 2 hours followed by centrifugation. The supernatant contains the nanoparticles.

Reaction quenching To quench the stain etching of 3C-SiC 0.04 mol CuF_2 (analytical grade, Reanal, Hungary) was added to 2 g SiC, 30 ml HF:HNO_3 3:1 solution. The result was checked by measuring the luminescence of the sample. For control experiment 0.04 mol CuF_2 was added to 2 g etched sample before removing the acid and accumulated overnight. The emission of SiC NPs at 450 nm was measured without adding CuF_2 in the reaction or with adding CuF_2 after the reaction. However, no PL signal was detected if CuF_2 was added before the reaction.

Synthesis of 6H-SiC NPs 6H-SiC NPs were prepared from hexagonal SiC powder (Sigma) using HF and $K_2S_2O_6$. 2.5 g SiC powder was ground in an alumina mortar and placed in the acid digestion vessel. 30 ml HF and 0.5g of $K_2S_2O_6$ was added to the SiC powder. The reaction was

carried out at 150 °C for 2 hours. For control experiments hexagonal SiC and 3C-SiC powder was reacted with HF:HNO_3, HF:$K_2S_2O_6$, HF:$K_2S_2O_8$, HF: HNO_3:$K_2S_2O_{8:}$ and distilled water. After reaction, the acid was removed and the sample was sonicated. After sonication, absorption and luminescence was measured to check for the presence of nanoparticles. In the case of 3C-SiC powder, we measured the already reported emission spectra after HF:HNO_3 and HF:$K_2S_2O_6$ treatment. In other cases, no PL signal was detected. In the case of 6H-SiC, we measured the emission peak at around 440 nm if and only if $K_2S_2O_6$ was applied. Figure S2 represents the Raman spectra and TEM images of the etched material. We emphasize here that the crystal structure of nanosized SiC is obscured both in the Raman spectra and HR-TEM images[8,9] caused by the quantum confinement effect and surface effects.

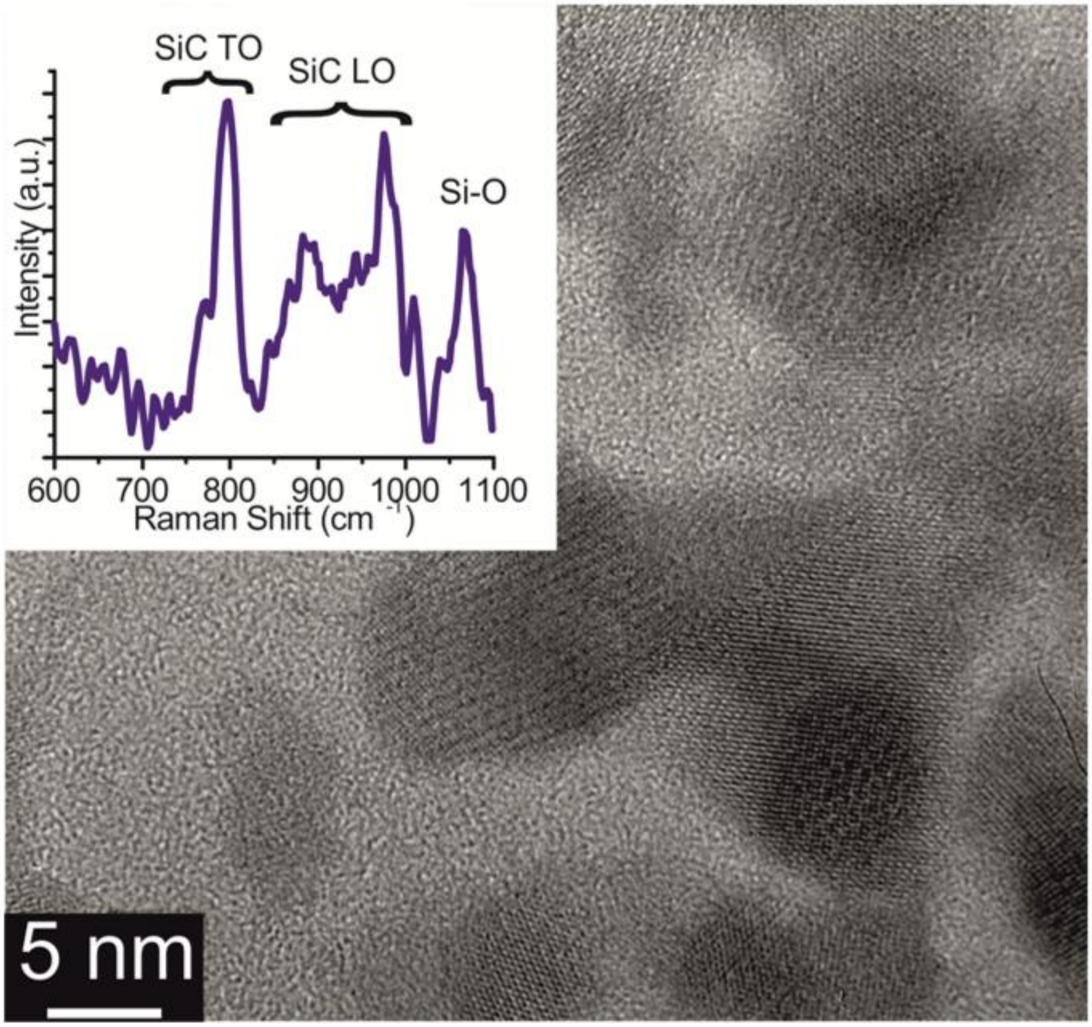

Fig. S2. Raman spectrum and HR-TEM images of nanostructures prepared by NPEGEC etching of 6H-SiC. TO labels the transverse optical (or planar optic) phonon mode of SiC while LO labels the longitudinal optical (or axial optic) phonon mode. Si-O represents the vibration of surface Si–O bonds. The HR-TEM images clearly demonstrate the formation of porous material after etching and the formation of nanoparticles.

Characterization of SiC SF-SiC and SiC NPs Microcrystalline powder of SiC was characterized by X-ray diffraction (HUBER G670 Guinier camera, Cu Kα radiation) prior to etching. Nanoparticles were characterized by high resolution electron microscopy (HRTEM - JEOL JEM-3010), UV-VIS absorption spectroscopy (Ocean Optics QE6500 spectrometer and Ocean Optics DH-2000-BAL light source), photoluminescence spectroscopy (Horiba Jobin Yvon NanoLog),Fourier transform infrared spectroscopy (Bruker Tensor 37), and Raman microscopy (Renishaw 1000 spectrometer and Leica DM/LM microscopy) with 514 nm excitation wavelength produced by a 0.6 mW Ar ion laser

Chemiluminescence measurements The chemiluminescence during stain etching of 3C-SiC was measured by using the Ocean Optics QE65000 spectrometer with 30 s integration time. An optical fiber was placed next to the PFA (perfluoroalkoxy polymer) tube containing SiC powder, HF, and HNO_3. The PFA tube together with the optical fiber was covered by aluminum foil and was buried under salt. Salt bath was used to heat up the reactor to about 100°C. Peaks were detected after 1 hour of heating. For control experiment HF:HNO_3 solution without SiC was also measured and no PL signal above the noise level was detected.

Calculation of stacking fault (SF) fraction in 3C-SiC SFs in 3C-SiC structure lead to locally hexagonal close packed (4H, 6H) layer sequences, without long-distance periodic order. Accordingly, the diffraction patterns only contain the distinct Bragg peaks of 3C-SiC phase (Fig. S4 A). The SFs cause broad diffraction features in the vicinity of the (111) reflection [10]. Quantitative analysis of these features requires sophisticated modeling of the layer sequences with fitted probabilities and correlations of the SF occurrences[11–13]. Instead of a potentially model-biased analysis we approximate the amount of stacking faults with the total diffracted intensity attributed to them that is normalized with the diffracted intensity of the 3C-SiC

crystalline phase. For this purpose we calculate the fraction $f_{\mathrm{SF}} = I_{\mathrm{SF}}/I_{\mathrm{CR}} = \sum(I_{\mathrm{obs}} - I_{\mathrm{calc}})/\sum(I_{\mathrm{calc}} - I_{\mathrm{bkg}})$, where I_{obs} is the measured X-ray powder pattern, I_{calc} is its single-phase 3C-SiC full profile fit and I_{bkg} is the instrumental background intensity. Both summations run over the points of the diffractogram in the 30–40 degrees angle range, where the scattering due to SFs is significant. f_{SF} changes monotonically with the Al content in the precursors, (Fig. S4 B), however, f_{SF} only approximates the real SF content (f_{SF} is the diffracted intensity fraction attributed to inhomogeneities in the crystal.

Calculation of distance between stacking faults (SF) The distance between two Si layers or two C layers in a SiC matrix is about 0.25 nm. If SFs appear quasi homogeneously with an average distance of 5 nm then this distance corresponds to 20 layers. If the average number of layers associated with single SFs is three[14] (see Fig. S1) then about 3/20≈15% is the ratio of SF layers vs. cubic SiC layers.

Connection between the stacking fault (SF) fraction and the size distribution Hexagonal inclusions in cubic SiC act as blocking layers for CB electrons that play a key role in controlling the size of the pore during stain etching. When a semiconductor is immersed in an electrolyte system, band bending takes place to equilibrate the Fermi level. Band bending at the nanoscale is size dependent and as the particle size decreases band bending decreases as well that slows down the electrochemical reaction. Below the size of the exciton Bohr radius band bending blocks the etching[15]. In other words, a certain minimum crystalline size is needed for migration of electrons and holes, in order to develop a depletion layer caused by the band bending effect (Figure S3A). A pore wall in the porous layer can be described as interconnected particles. As long as those particles are connected evenly to each other, electron and hole migration is possible and band bending is realized. When the particles are separated or the migration of carriers is hindered then

band bending depends only on the size of the separate particles. In this situation, the final particle size during the etching is determined by the exciton Bohr radius[16,17]. As the migration of the electrons is blocked by the hexagonal inclusions in cubic SiC, the resultant diameter of the SiC NPs should be around twice the exciton Bohr radius which is about 5 nm (Figure S3B-C) next to SF. As the SF concentration increases, the distance between two inclusions decreases and as a result, the population of SiC NPs with size close to the exciton Bohr radius increases (Fig S3B). By assuming uniformly distributed and ordered SFs (see in Fig. S1), with each SF consisting of three bilayers (small 4H-SiC inclusion), the average distance between two SFs is reduced to 5 nm at about 15% SF concentration, and under these circumstances, the size of the nanoparticles shall be equal to twice the exciton Bohr radius.

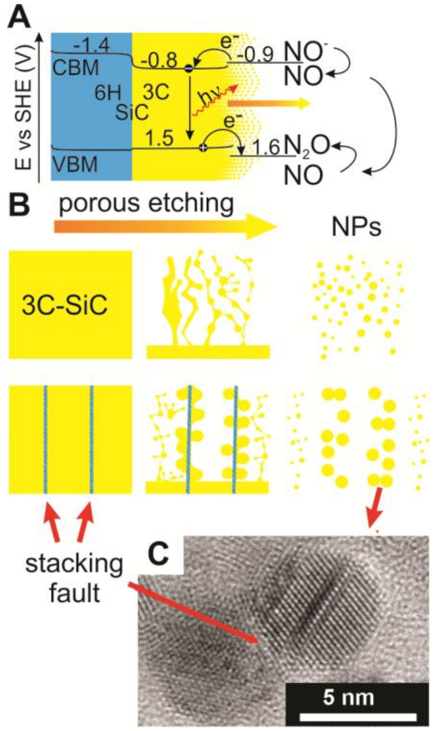

Fig. S3. The role of pore formation and nanoparticle synthesis in spatially varying CBM system. (A) Redox reactions during SiC etching in HF:HNO_3. (**B**) Pore formation with and without stacking faults and uniform particle synthesis in a spatially varying CBM system. Only the lower CBM region can contribute to the pore formation. (**C**) Transmission electron microscope image of two particles connected by a hexagonal inclusion. The hexagonal inclusions in SiC are nearly two-dimensional layers that contribute to the uniform size synthesis. When two particles were formed by the etching process on the opposite side of the SF then the particles are not separated after sonication.

SiC with different SF fraction was prepared by varying the Al content in the precursor mixture between 0.5-5 at%. The presence of SFs can be observed in XRD spectrum (Figure S4 A). Figure S4 B shows that the SF fraction increases together with adding additives. Nanoparticles produced from these materials were characterized by photoluminescence spectroscopy. Figure S4 C shows the PL spectra of NPs synthesized from SiC with different SF content. The broad

emission peak can be fitted with two Gaussians. The peak at around 450 nm corresponds to the smaller particles (1-4 nm) while the peak around 530 nm corresponds to the larger particles (4-6 nm) as plotted in Fig. S4 D. By increasing the SF fraction the PL intensity at around 530 nm also increases.

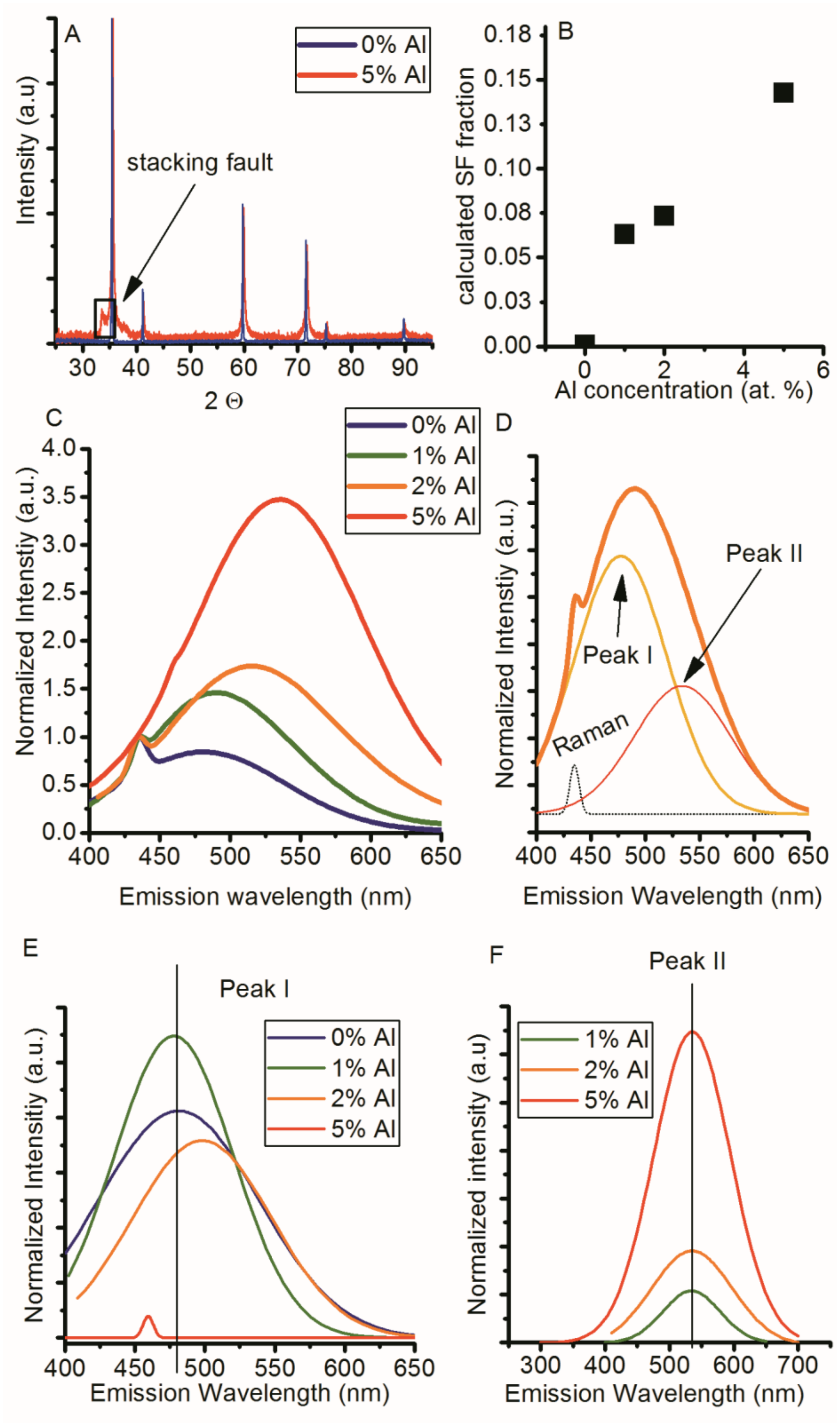

Fig. S4 (A) XRD of SiC synthesized with adding Al to the precursors. The black box shows the signature of hexagonal layers or stacking faults (SF). **(B)** Calculated SF fractions vs. Al

concentration in the precursors. **(C)** PL of SiC NPs prepared from SiC with various SF fractions (Exc: 380 nm). Increasing SF fraction in the 3C-SiC host matrix results in a redshift in the emission spectra of the synthesized NPs. **(D-F)** Gaussian fit of the emission spectra. Peak I corresponds to the PL signature without Al in the precursor. Peak II only appears when Al is present in the precursor. Redshift between these peaks occurs because of the increasing concentration of larger particles [18].

Data source for Figure 3 VBM and CBM energies are taken from the literature. Electronic structure of nanowires is in the center of intense research, however, the interpretation of experimental data is often difficult. We used preferentially well-confirmed experimental data when available or results from *ab initio* calculations. References: Si [19], Ge [20,21], InP, GaN [22,23], ZnO [24,25].

Note for redox potential values:

All redox potential data taken from literature and reported here are measured at room temperature. The redox potential shifts upward by a few tens of millielectron-volts at elevated temperatures (~80 °C).

References

1. Fan, J. & Chu, P. K. *Silicon Carbide Nanostructures*. (Springer International Publishing, 2014). doi:10.1007/978-3-319-08726-9
2. Patrick, L., Hamilton, D. R. & Choyke, W. J. Optical properties of cubic SiC: Luminescence of nitrogen-exciton complexes, and interband absorption. *Phys. Rev.* **133,** A1163 (1964).
3. Davydov, S. Y. On the electron affinity of silicon carbide polytypes. *Semiconductors* **41,** 696–698 (2007).
4. Lindefelt, U., Iwata, H., Öberg, S. & Briddon, P. R. Stacking faults in 3C-, 4H-,- 6H-SiC polytypes investigated by an *ab initio* supercell method. *Phys. Rev. B* **67,** 155204 (2003).
5. Mukasyan, A. S., Lin, Y. C., Rogachev, A. S. & Moskovskikh, D. O. Direct combustion synthesis of silicon carbide nanopowder from the elements. *J. Am. Ceram. Soc.* **96,** 111–117 (2013).

6. Dravecz, G., Bencs, L., Beke, D. & Gali, A. Determination of silicon and aluminum in silicon carbide nanocrystals by high-resolution continuum source graphite furnace atomic absorption spectrometry. *Talanta* **147,** 271–275 (2016).

7. Beke, D. *et al.* Preparation of small silicon carbide quantum dots by wet chemical etching. *J. Mater. Res.* **28,** 44–49 (2013).

8. Botsoa, J. *et al.* Photoluminescence of 6H–SiC nanostructures fabricated by electrochemical etching. *J. Appl. Phys.* **102,** 83526 (2007).

9. Guo, X., Dai, D., Fan, B. & Fan, J. Experimental evidence of α → β phase transformation in SiC quantum dots and their size-dependent luminescence. *Appl. Phys. Lett.* **105,** 193110 (2014).

10. Pujar, V. V. & Cawley, J. D. Effect of Stacking Faults on the X-ray Diffraction Profiles of beta-SiC Powders. *Journal of the American Ceramic Society* **78,** 774–782 (1995).

11. Pujar, V. V & Cawley, J. D. Computer Simulations of Diffraction Effects due to Stacking Faults in β-SiC: I, Simulation Results. *J. Am. Ceram. Soc.* **80,** 1653–1662 (2005).

12. Pujar, V. V & Cawley, J. D. Computer simulations of diffraction Effects due to Stacking Faults in β-SiC: II: Experimental Verification. *J. Am. Ceram. Soc.* **84,** 2645–2651 (2001).

13. Ungár, T. Characterization of nanocrystalline materials by X-ray line profile analysis. *J. Mater. Sci.* **42,** 1584–1593 (2007).

14. Iwata, H. Stacking faults in silicon carbide. *Phys. B Condens. Matter* **340–342,** 165–170 (2003).

15. Kolasinski, K. W. Charge transfer and nanostructure formation during electroless etching of silicon. *J. Phys. Chem. C* **114,** 22098–22105 (2010).

16. Hagfeldt, A. & Graetzel, M. Light-Induced Redox Reactions in Nanocrystalline Systems.

Chem. Rev. **95,** 49–68 (1995).

17. Wang, F. & Buhro, W. E. Determination of the rod-wire transition length in colloidal indium phosphide quantum rods. *J. Am. Chem. Soc.* **129,** 14381–14387 (2007).
18. Beke, D., Szekrényes, Z., Czigány, Z., Kamarás, K. & Gali, Á. Dominant luminescence is not due to quantum confinement in molecular-sized silicon carbide nanocrystals. *Nanoscale* **7,** 10982–10988 (2015).
19. Memming, R. R. Photoinduced charge transfer processes at semiconductor electrodes and particles. *Electron Transf. I* **169,** 105–181 (1994).
20. Kaewmaraya, T. & M., A. Structural and electronic properties of Ge polytype junctions from an ab-initio perspective. in *2016 Spring : Symposium O | EMRS* (2016).
21. Ikonic, Z., Srivastava, G. P. & Inkson, J. C. Optical properties of twinning superlattices in diamond-type and zinc-blende-type semiconductors. *Phys. Rev. B* **52,** 14078–14085 (1995).
22. Stampfl, C. & Van de Walle, C. Energetics and electronic structure of stacking faults in AlN, GaN, and InN. *Phys. Rev. B* **57,** R15052–R15055 (1998).
23. Faria Junior, P. E. & Sipahi, G. M. Band structure calculations of InP wurtzite/zinc-blende quantum wells. *J. Appl. Phys.* **112,** (2012).
24. Khranovskyy, V. *et al.* Crystal phase engineered quantum wells in ZnO nanowires. *Nanotechnology* **24,** 215202 (2013).
25. Asthana, A., Momeni, K., Prasad, A., Yap, Y. K. & Yassar, R. S. On the correlation of crystal defects and band gap properties of ZnO nanobelts. *Appl. Phys. A Mater. Sci. Process.* **105,** 909–914 (2011).
